# A bibliometric analysis of online faculty professional development in higher education

**DOI:** 10.1186/s41039-022-00196-w

**Published:** 2022-05-16

**Authors:** Yanjun Gao, Su Luan Wong, Mas Nida Md. Khambari, Nooreen Noordin

**Affiliations:** 1 Universiti Putra Malaysia, 43400 Serdang, Selangor Malaysia; 2 Anyang Institute of Technology, Anyang, 455000 China; 3 Department of Science and Technical Education, Faculty of Educational Studies, Universiti Putra Malaysia, 43400 Serdang, Seri Kembangan, Selangor Malaysia

**Keywords:** Professional development, Faculty development, Online faculty, Online faculty professional development

## Abstract

Research on online faculty professional development (OFPD) in higher education has increased in recent years. As there is, nevertheless, a scarcity of quantitative investigations on research publications in this area, a bibliometric analysis of 248 publications collected from the Scopus database was conducted. Biblioshiny and VOSviewer software tools were used for descriptive and network analyses. The research results showed that the overall trend of publication in this domain increased steadily at an annual growth rate of 14.11% during the past 25 years. *Journal of Asynchronous Learning Network* and *Computers and Education* ranked the highest among journals with regard to publication number and citation number, respectively. With a total of 298 citations to his paper, Peter Shea was ranked the most impactful author while Maria Northcote, with five publications, was the most productive. In terms of geographical location of research activity, America played the leading role, with Asia emerging in this field. The publication entitled “*A Research Agenda for Online Teacher Professional Development*” by Dede et al. topped the list for both total citations and average yearly citations. As to recent trends, teacher professional development through online teaching was emergent partly due to the outbreak of Covid-19. Pedagogy training, online community building, and facilitating online teachers were the themes that researchers favored. The study will contribute toward better understanding of the existing literary landscape of research on OFPD given the potential of OFPD in enhancing faculty’s effectiveness in their classrooms and over the course of their teaching careers.

## Introduction

Online education has gained increasing attention in higher education institutions in recent decades (Leary et al., 2020), partly due to its unique advantages such as accessibility, affordability, and flexibility (Dhawan, 2020). Compared with declining campus enrollment, online learning enrollment has been growing consistently over the years. In the USA, for instance, 6,359,121 college students enrolled in at least one online course in a semester in the fall of 2016, with the percentage of online student enrollment increasing by 5.6% compared with that of the previous year, and accounting for 31.6% of all students (Seaman et al., 2018). In response to the changing educational modality, many institutions consider online education as an important part of their long-range development strategies (Eichelberger & Leong, 2019). Subsequently, a series of courses and degree programs that are offered by online delivery and online education have moved into an established institutional function from an experimental phase (Legon & Garrett, 2017).

The expanding number of online students and online courses in higher education has created a huge demand for online teachers who play a critical role in the successful implementation of online education (Baran & Correia, 2014). Owing to the current global Covid-19 pandemic, an unprecedented number of traditional courses have been hastily transferred to an online mode to keep social distance (Andel et al., 2020). Teaching online is evidently different from teaching in a traditional environment in terms of pedagogical, social, managerial, and technical competencies (Guasch et al., 2010; Smith, 2005; Williams, 2003). It does not guarantee that teachers with rich teaching experience and advanced skills in using technology will automatically become successful e-instructors (Gulbahar & Kalelioglu, 2015). Instead, it is highly possible that what is effective or feasible for them in a traditional face-to-face teaching setting may not work in an online setting (McQuiggan, 2012). Therefore, it is essential for teachers to adopt new pedagogies, develop the requisite competencies and reconstruct their persona as online teachers (Adnan, 2018). Nevertheless, misunderstanding or even confusion may occur for faculty members, both in adjusting roles and adopting new skills during the transition (Pierce-Friedman & Wellner, 2020) especially when they are confronted with online teaching for the first time (Brookfield, 2015). In the absence of a faculty benchmark for online teaching since it is a new strategy, it would not be surprising if teachers taught as they themselves had been taught previously, i.e., like in the traditional classroom (Schmidt et al., 2016). It is less possible for faculty to know intuitively how to conduct online teaching effectively (Polloff & Pratt, 2001). Hence, it is vital for institutions to create effective professional development opportunities to support faculty’s transition and training for novices, or even veterans, to teach online effectively (Mohr & Shelton, 2017; Schmidt et al., 2016).

Research on OFPD has been on the rise, both theoretically and empirically. There has been extensive research recently on what effective practices of OFPD should be. According to McQuiggan (2012), faculty should be regarded as adult learners. With this perspective, the characteristics of faculty as adult learners need to be considered, including their educational background, life experiences, and learning preferences. According to Elliott et al. (2015), flexibility and self-paced scheduling contribute to successful professional development, and faculty should receive training which is aligned with their instructional context. In terms of the best practice framework for professional development of online faculty, Mohr and Shelton (2017) gained consensus of the essential elements of the online faculty professional development from a panel of experts with a four-survey-round Delphi Method. The findings identified two categories, namely essential professional development and institutional strategies, both of which are significant to the new and existing professional development plans. A holistic framework was proposed by integrating organization, community, and teaching levels to support teaching in an online environment (Baran & Correia, 2014). Based on the aforesaid framework of Baran and Correia (2014), Martin et al. (2019) conducted a study on the professional development support among US and German instructors. The results showed that faculty’s needs could be met by support from administration, individuals, pedagogy, and technology.

Growing interest in online courses highlights the important status of OFPD in the eyes of the academic community. To have a better understanding of this academic discourse, it is necessary to look into the publications related to OFPD in their entirety. However, the existing literature review on teacher professional development has not targeted OFPD sufficiently (Philipsen et al., 2019). Furthermore, based on the author’s observation, studies have rarely been conducted to explore the overall literature from a global perspective in terms of distribution patterns, the most influential contributors, current development hotspots, and future research trends in this field.

Given the gap in literature, it would be quite pertinent to conduct a comprehensive review covering the recent decades to look into the relevant publications. Based on the aforementioned reasons, a bibliometric analysis was carried out by evaluating and mapping the relevant literature in the field of OFPD in higher education. An extensive look into the existing publications on OFPD will be presented by addressing the following three research questions:

1. What is the distribution pattern of annual published documents on OFPD in higher education?
2. What or who are the main contributors in terms of journals, authors, countries, and documents on OFPD in higher education?
3. What are the most frequently discussed themes and the corresponding evolving trends on OFPD in higher education?

## Methodology

A bibliometric analysis was conducted to look into the published output relevant to OFPD. This formed a structural image of publications by measuring and analyzing bibliographic data published in the specific domain (Zeng & Chini, 2017).

### Research design

The research design adopted mainly involved identifying appropriate bibliographic databases, developing search criteria, and selecting analysis software tools.

Scopus database was considered appropriate for the study based on the following reasons: 1) It provided relevant and reliable information on publications with its bibliographic multidisciplinary data and its policy of prioritizing the peer review procedure. 2) Scopus’ 20% wider coverage in time compared with the Web of Science, a frequently used database in a bibliometric analysis, was an advantage in conducting evolution and citation analysis (del Río-Rama et al., 2020). 3) All authors were contained in cited references, allowing it to be more accurate in conducting author-based analyses (Zupic & Cater, 2015). Moreover, the data could be exported directly from Scopus in a format supported by most software used for bibliometric analysis.

Research criteria were set according to the research objectives and research questions. First of all, the search words that focused on both “online teachers” and “professional development for online teachers” were identified. Building on previous research, the words occurring in the relevant articles frequently related to teachers in an online environment were established: “teaching online”, “teach online”, “online instructor”, “online faculty”, “online teacher”, and “online teaching”. Likewise, the query words occurring in the articles related to professional development were identified. This included “professional development”, “teacher development”, “teacher training”, “faculty development”, “instructor development”, “online faculty development” and “faculty development programs”. Finally, the Boolean operators “OR” and “AND” were used to connect these search words to broaden the search and to obtain as many relevant results as possible, while excluding irrelevant data.

The time of publication was not limited to any specific year so that the whole evolution of OFPD over time could be assessed. All subject areas were included since the study was multidisciplinary and cross-disciplinary. Considering the source types of the publication, only peerreviewed journal articles were included because peer review procedures were considered to produce more reliable results (Niñerola et al., 2019). Only publications in the English language were included in this search.

Biblioshiny and VOSviewer were selected as software tools in this study. Biblioshiny analyzes broad categories ranging in the analytics and graphs from individual contributions to the social network, with bibliographic data extracted from the Scopus database (Moral-Muñoz et al., 2020). VOSviewer is a powerful tool to map and visualize network structure with bibliographic data from many databases such as Web of Science and Scopus (Moral-Muñoz et al., 2020).

### Data collection

Based on the research criteria set in the research design phase, the whole search strategy is summarized in Table 1.

**Table 1:** Search strategy

Type	Criteria
Database	Scopus
Search string	TITLE-ABS-KEY ("teaching online" OR "teach online” OR “online instructor” OR “online faculty” OR “online teacher” OR “online teaching” AND “professional development” OR “teacher development” OR “teacher training” OR “faculty development” OR “instructor development” OR “online faculty development" OR “online faculty training” OR “faculty development programs”)
Time span	All
Subject area	All
Document type	Article
Source of the publications	Journal
Language	English
Search date	April 25, 2021

**Fig. 1:**
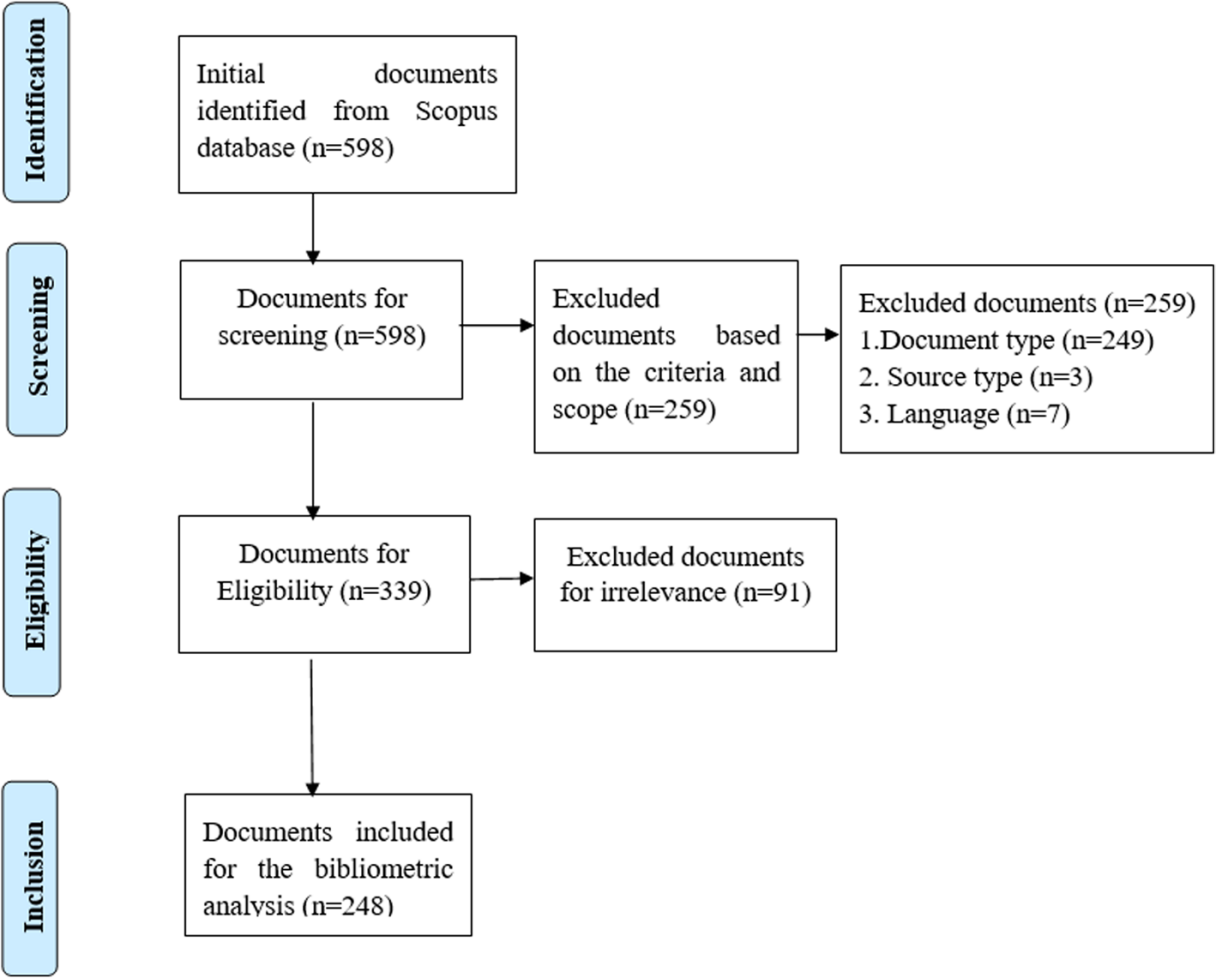
PRISMA method procedure for identifying and selecting the documents

A total of 598 publication results were initially captured according to the search keywords. Of these, 259 publications were excluded for violating the criteria for the research design including the document type (249), the source type of the publication (3), and language (7) at this stage. The remaining 339 publications were manually checked for eligibility by examining the titles and the abstract of each article, following which a total of 91 publications were excluded for irrelevance to the study, because these articles all focused on teachers’ professional development by online mode instead of focusing on professional development for online teachers. After the elimination of duplicates, unidentified bibliometrics, and irrelevance, the final 248 records were exported in “CSV” (comma-separated value) format from the Scopus database for use in the subsequent bibliometric analysis. The whole tracking process of data collection was carried out using the Preferred Reporting Items for Systematic Reviews and Meta-Analysis (PRISMA) method with a four-phase flow diagram (Liberati et al., 2009) as shown in Fig. 1.

### Data analysis

Descriptive analysis was conducted to describe the number of publications, whole development trend, citations and productivity. Impact of the authors, articles, journals, countries, and the analysis of the author keywords, and the topic trends were also examined. Network analysis was carried out by data visualization and scientific mapping consisting of the 1) analysis of the co-occurrence of keywords; 2) cluster analysis; and 3) thematic evolution analysis.

## Bibliometric analysis of literature: results

The bibliometric analytic results are presented here based on the three research questions.

### What is the distribution pattern of published annual documents on OFPD in higher education?

There was a total of 248 articles on OFPD from 134 sources in the Scopus database in the last 25 years from 1997 until the present, with an average of 13.21 citations per document. Out of a total of 608 authors, 60 authors were responsible for single-authored documents; 90% of the documents were authored by more than one author. In terms of author collaboration, there was an average of 2.71 authors per document, generating a collaboration index of 2.96. Meanwhile, it was observed that 401 Keywords Plus, words or phrases frequently appearing in the titles of an article's references, in the title of the article itself, and 598 Author's Keywords were developed from the collected data. The information collected is summarized in Table 2.

**Table 2:** Main information of data collected

Description	Results
Timespan	1997–2021
Sources	134
Articles	248
Average citations per document	13.21
Keywords plus	401
Author's keywords	598
Authors	608
Authors of single-authored documents	60
Authors of multi-authored documents	548
Single-authored documents	63
Documents per author	0.408
Authors per document	2.45
Co-authors per document	2.71
Collaboration index	2.96

**Fig. 2:**
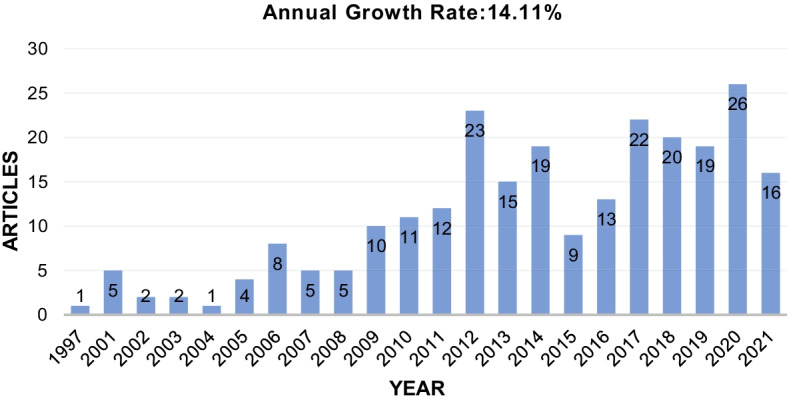
Annual production of OFPD publications from 1997 to 2021

The first publication on OFPD by Briano et al. (1997) focused on how the computer facilitated online teacher training, especially with regard to communication in environmental education. Since then, the overall trend of publication has maintained a stable increment with an annual growth rate of 14.11%, despite some fluctuations in publication numbers over the past 25 years. Overall, the developmental trend could be divided into three stages. In the initial stage (1997–2008), there was only one publication in 1997 and none in the following 3 years. The annual publication rate was 2.75 per year at this initial stage. In the second stage (2009–2016), the quantity grew gradually with an average of 14 publications annually. The number of publications reached its peak in 2012 with 23 publications. However, there was a slight declining trend in 2015 and 2016, with 9 and 13 publications, respectively. Research on OFPD saw rapid development with more than 20 annual publications at the third stage (2017–2021). A total of 16 journal articles were already published during the first 4 months of 2021, so it can be predicted that 2021 will be a productive year for OFPD publication. The annual production of OFPD publications is shown in Fig. 2.

### What or who are the main contributors in terms of journals, authors, countries, and documents on OFPD in higher education?

#### Contribution of journals in terms of productivity and impact

**Fig. 3:**
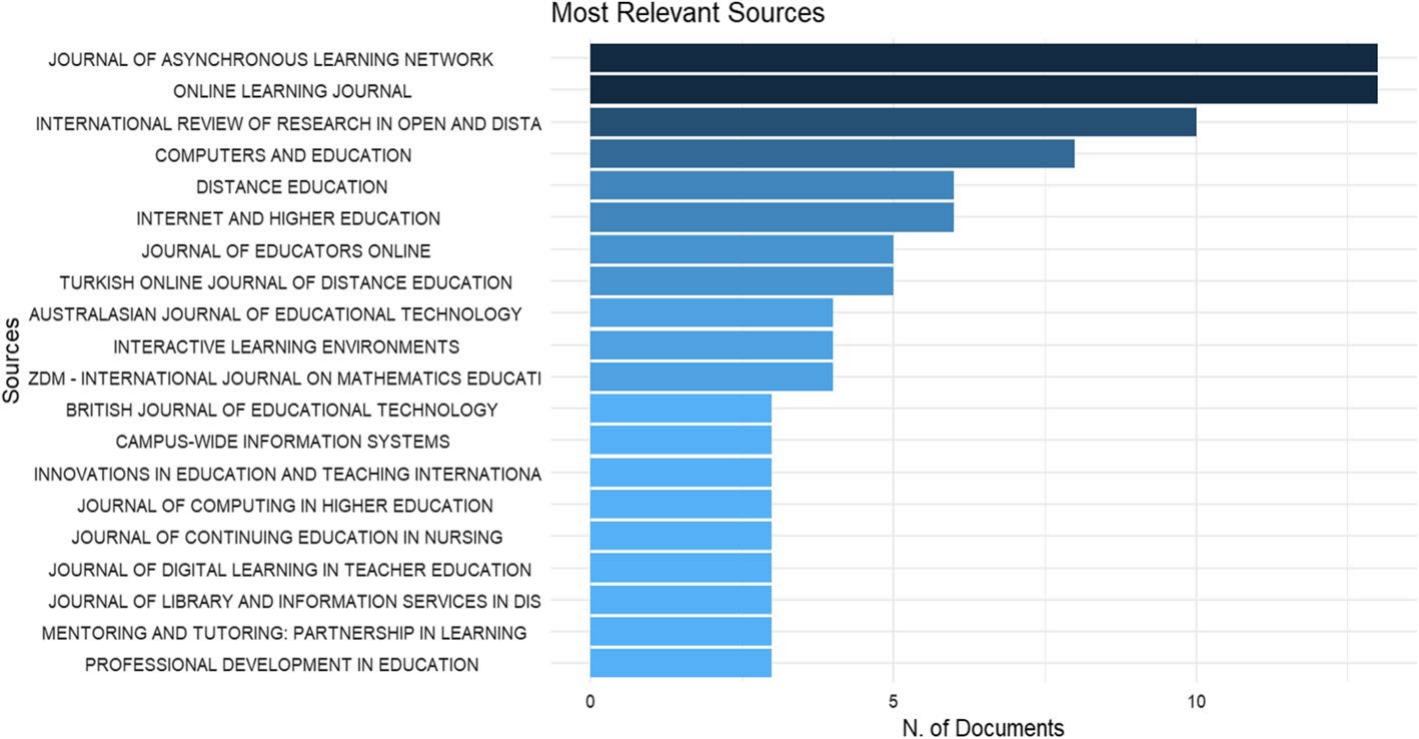
Most relevant sources for OFPD in terms of number of publications

The top 20 journals out of the total 134 in terms of the number of publications are shown in Fig. 3. *Journal of Asynchronous Learning Network, Online Learning Journal, and International Review of Research in Open and Distance Learning* ranked top three with publication numbers of 13, 13, and 10, respectively. There were 45 journals out of 134 that published more than one article related to OFPD.

It is also important to know the source impact besides productivity. Hence, the indicator of citation, as one of the most common methods to assess the influence of authors, articles, or journals (Garfield, 1972), was used to look into the influence of the journals under study. The top seven most cited sources which were cited more than 100 times were *Computers and Education* (420), *Journal of Teacher Education* (334), *Journal of Asynchronous Learning Network* (248), *Distance Education* (221), *Internet and Higher Education* (176), *Teaching and Teacher Education* (175) and *International Review of Research in Open and Distance Learning* (149). The h-index which can evaluate both citation impact and productivity of the production (Hirsch, 2005) was looked at to measure source impact in terms of productivity and citation impact. *Journal of Asynchronous Learning Network* and *International Review of Research in Open and Distance Learning* were the top two journals according to the h-index indicator. Information of Source impact is shown in Table 3.

**Table 3:** Source impact

Source	h_index	TC	NP	PY_start
Computer and Education	6	420	8	2009
Journal of Teacher Education	2	334	2	2009
Journal of Asynchronous Learning Network	9	248	13	2012
Distance Education	5	221	6	2002
Internet and Higher Education	6	176	6	2002
Teaching and Teacher Education	3	175	3	2013
International Review of Research in Open and Distance Learning	7	149	10	2001
Journal of Educators Online	4	81	5	2011
Online Learning Journal	6	79	13	2016
Interactive Learning Environments	2	67	4	2008

TC, total citation; NP, number of publications; PY, publication year

**Fig. 4:**
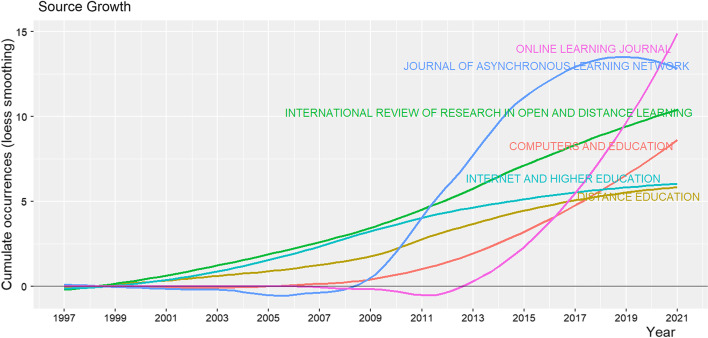
*Source* dynamics

It is also important to look into evolution of the sources related to professional development for online teaching. Six journals were analyzed for cumulative occurrences as shown in Fig. 4. Overall, the growth trends in publications indicated that there were few publications initially. *International Review of Research in Open and Distance Learning, Internet and Higher Education, Distance Education* grew at a stable rate over the whole period. On the other hand, *Computers and Education, Journal of Asynchronous Learning Network,* and *Online Learning Journal* developed rapidly in publication numbers since its first publication. Within a relatively short time, the *Journal of Asynchronous Learning Network* had published 13 articles from 2012 to 2021, as had *Online Learning Journal* from 2016 to 2021—making them the top journals in terms of the number of publications.

#### Contribution of authors in terms of productivity and impact

The authors’ productivity distribution regarding articles frequency published on OFPD was measured using Lotka`s Law. According to the results of Lotka’s Law, 556 authors out of a total of 608 authors (91.4%) published one article in the field of online faculty professional development; 45 authors (7.4%) published two papers; two authors (0.3%) published three papers; four authors (0.7%) published four papers and one author (0.2%) published five papers, the highest in publication number.

As shown in Table 4, the top authors in terms of the number of the published papers were Northcote, M. (5), Gosselin, K. P. (4), Kilgour, P. (4), Meyer, K. A. (4), and Reynaud, D. (4). However, the productivity of the authors in terms of publication number was not equal to the impact they generated in terms of citation number. To identify the most impactful or influential authors in the study field, citation analysis of authors was undertaken (Table 4). The results showed that the top 10 authors out of 608 received more than 100 citations for their publications. With a total of 298 citations, Shea, P. was ranked the most impactful author in the field of professional development for online education. Mccloskey, E.M. followed Shea, P. with 274 citations from two publications.

**Table 4:** Authors’ productivity and impact

First author	TC	NP	PY_start	First author	TC	NP	PY_start
Shea, P	298	2	2005	Morthcote, M	31	5	2015
Mccloskey, E. M	274	2	2009	Gosselin, K. P	28	4	2015
Breit, L	262	1	2009	Kilgour, P	28	4	2015
Dede, C	262	1	2009	Meyer, K. A	57	4	2014
Ketelhut, D. J	262	1	2009	Reynaud, D	28	4	2015
Whitehouse, P	262	1	2009	Adnan, M	23	3	2016
Bidjerano, T	238	1	2009	Rienties, B	168	3	2013
Rienties, B	168	3	2013	Aleger de la rosa, O. M	9	2	2007
Brouwer, N	155	2	2013	Aleger, O. M	18	2	2006
Lygo-baker, S	155	2	2013	Anderson, M	17	2	2015

TC, total citations; NP, number of publications; PY, publication year

It is worth noticing that many authors received a higher number of citations with fewer publications. For example, the co-authors Breit, Dede, Ketelhut, and Whitehouse received 262 citations from only one piece of work, which meant their paper was particularly impactful in the field of study.

#### Contribution of countries in terms of productivity and impact

The productivity of publications is an index that reflects the development of a country in a specific academic field (Xie et al., 2020). In this study, a total of 38 countries were identified as having conducted OFPD research during 1997–2021. The USA was the most prolific country with 323 publications, followed by Australia (46), UK (43), China (22), Spain (20), India (16), Turkey (15), Malaysia (14), Netherlands (13) and Canada (12). From the geographical distribution of scientific distribution, therefore, North America played a leading role with 335 documents, followed by Europe (76), Asia (67), and Oceania (46). The exclusion of seven non-English language articles notwithstanding, North America, and especially USA, were the most active regions undertaking OFPD research, with other regions like Europe, Asia and Oceania also contributing substantially.

As mentioned above, productivity does not necessarily equal impact. Hence, the analysis of total citations and average article citation was performed to assess the country's impact in the field of interest. It can be seen from Table 5 that the publications from the USA drew the most citations, totaling 848, followed by the United Kingdom with 373 and Australia with 224 total citations. As to the average number of article citations, Georgia, the United Kingdom, and China were the top three leaders with 44.00, 26.64, and 23.86 citations, respectively. The top ten countries responsible for the highest impact in terms of total citations were also the highest in average article citation compared with the other 28 countries.

**Table 5:** Countries producing the highest impact in terms of total citations and average article citations

Country	Total citations	Country	Average article citations
USA	848	Georgia	44.00
United Kingdom	373	United Kingdom	26.64
Australia	224	China	23.86
China	167	India	23.67
Georgia	88	Australia	20.36
India	71	Switzerland	20.00
Turkey	59	Norway	18.00
Norway	54	USA	15.42
Ireland	44	Turkey	14.75
Switzerland	40	Ireland	14.67

#### Contribution of documents in terms of impact

Citation analysis was undertaken to look into the most influential documents in the field of OFPD. It was observed that a total of 208 articles out of 248 articles received more than one citation, which meant that about 90% of documents were cited more than once. The top ten most cited documents that were identified had more than 50 citations for each document (Table 6). The article entitled “*A Research Agenda for Online Teacher Professional Development”* published by Dede et al. in 2009 sat at the top with respect to both total citations and average yearly citations. Similar highly cited documents can be regarded as important references in the field of professional development for online teaching.

**Table 6:** Top ten most globally cited OFPD documents

First author	PY	Title	Total citations	TC per year
Dede, C. J	2009	A Research Agenda for Online Teacher Professional Development	262	20.15
Shea, P	2009	Community of inquiry as a theoretical framework to foster “epistemic engagement” and “cognitive presence” in online education	238	18.30
Rienties, B	2013	The effects of online professional development on higher education teachers' beliefs and intentions towards learning facilitation and technology	136	15.11
Tseng,F–C	2014	A study of social participation and knowledge sharing in the teachers' online professional community of practice	105	13.13
King, K. P	2002	Identifying success in online teacher education and professional development	90	4.50
Bawane, J	2009	Prioritization of online instructor roles: implications for competency‐based teacher education programs	71	5.46
Sims, R	2002	Enhancing Quality in Online Learning: Scaffolding Planning and Design Through Proactive Evaluation	68	3.40
Shea, P	2005	Increasing Access to Higher Education: A study of the diffusion of online teaching among 913 college faculty	60	3.53
Gautreau, C	2011	Motivational Factors Affecting the Integration of a Learning Management System by Faculty	52	4.72
Hou, H-T	2009	Using blogs as a professional development tool for teachers: analysis of interaction behavioral patterns	52	4.00

TC, total citations; PY, publication year

### What are the most frequently discussed themes and the corresponding evolving trends on OFPD in higher education?

This section examines the most frequent themes in OFPD research and evolving trends over time by looking into the keywords and the thematic evolution.

#### Most frequently discussed themes

Keywords are taken as an elevated summarization and refinement of the scientific publication (Xie et al., 2020). A co-occurrence analysis of keywords is an efficient way to illustrate the structure of scientific knowledge, explore hotspots and uncover patterns in a specific field (Aria & Cuccurullo, 2017; Su et al., 2019). By mapping and clustering the terms extracted from keywords, research themes can be determined concisely (Xie et al., 2020).

**Fig. 5:**
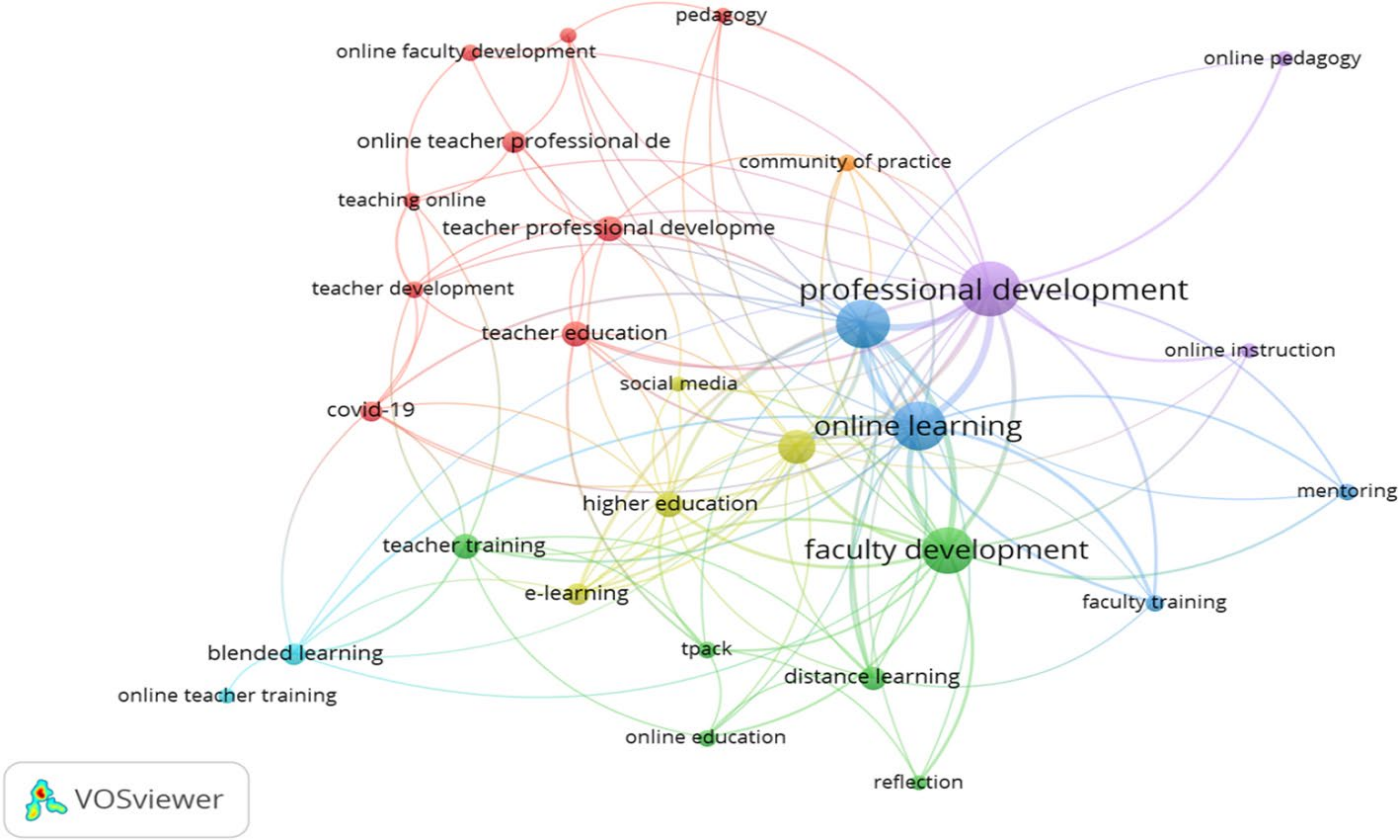
Co-occurrence network of author keywords

As shown in Fig. 5, the mapping comprised circles and lines with each circle representing a node that referred to an author keyword. The size of the circle reflected the occurrence frequency, i.e., larger circles stood for more times that the keywords appeared in the literature. The line between two nodes demonstrated the strength of the relationship of two keywords. Here, the thickness of the line between two nodes indicated the frequency of co-occurrence of two keywords while the distance between two nodes indicated the strength of topic similarity. The color of each circle corresponded to an individual cluster of the keyword where the node belonged.

In this part of the study, the co-occurrence of author keywords was used as an analysis unit to look into the research themes in the field of OFPD. A minimum threshold of the co-occurrence was set at four; then, the co-occurrence of a total of 29 author keywords from 248 publications were visualized and then further categorized into different categories. Figure 5 shows that the author keywords were grouped into seven different clusters from the coloration of the nodes.

Cluster 1 consisted of a grouping of keywords that included “covid-19”, “instructional design”, “online faculty development”, “online teacher professional development”, “pedagogy”, “teacher development”, “teacher education”, “teacher professional development”, and “teaching online”. This cluster had the most items and it represented the latest research theme focused on how professional development helped faculty to conduct online teaching during the global Covid-19 pandemic outbreak.

Cluster 2 consisted of a grouping of keywords that included “distance learning”, “faculty development”, “online education”, “reflection”, “teacher training”, and “tpack”. TPACK (Technological Pedagogical Content Knowledge) was given much attention by researchers to explore online faculty professional development.

Cluster 3 consisted of a grouping of keywords that included “faculty training”, “mentoring”, “online teaching”, and “online teaching”. The theme focused on the facilitation provided for online education.

Cluster 4 consisted of a grouping of keywords that included “distance education”, “e-learning”, “higher education”, and “social media”. The theme was more related to social media-support for online teaching and learning.

Cluster 5 consisted of a grouping of keywords that included “online instruction”,

“online pedagogy”, and “professional development”. The theme “pedagogy” showed the urgent need for pedagogy content for online teachers.

Cluster 6 consisted of a grouping of keywords that included “blended learning” and “online teacher training”. The theme was much about the blended learning mode.

Cluster 7 consisted of a grouping of keywords that included “community of practice”, which stressed the importance of building a community of practice.

#### Thematic evolution

In order to present the thematic development and evolution of the theme of OFPD over time, the analysis of thematic evolution was carried out, and the results were plotted in a thematic diagram. Four typologies of themes can be defined in the diagram according to the Callon centrality and Callon density. Callon centrality indicates the extent of the importance of a specific topic involved in the whole collection, while Callon density indicates the extent of development of a specific topic (Aria et al., 2020).

Specifically, if these themes are in the upper-right quadrant, it indicates that the themes are very important and well developed in the whole study field because they feature both high centrality and high density. Likewise, the themes in the lower-right quadrant are very important in one domain for their high centrality while focusing on specific transversal topics to a study field for their low density. If the themes lie in the lower-left quadrant, it indicates that they are marginal and less developed for both low centrality and low-density, whereas themes in the upper-left quadrant are of relatively weak importance, with low centrality but well developed in one specific domain with high density (Aria et al., 2020).

In this study, the temporal interval (1997–2021) was divided into three sub-periods, viz. from 1997 to 2008, from 2009 to 2016, and from 2017 to 2021. Author keywords were used as analysis units in each sub-period.

**Fig. 6:**
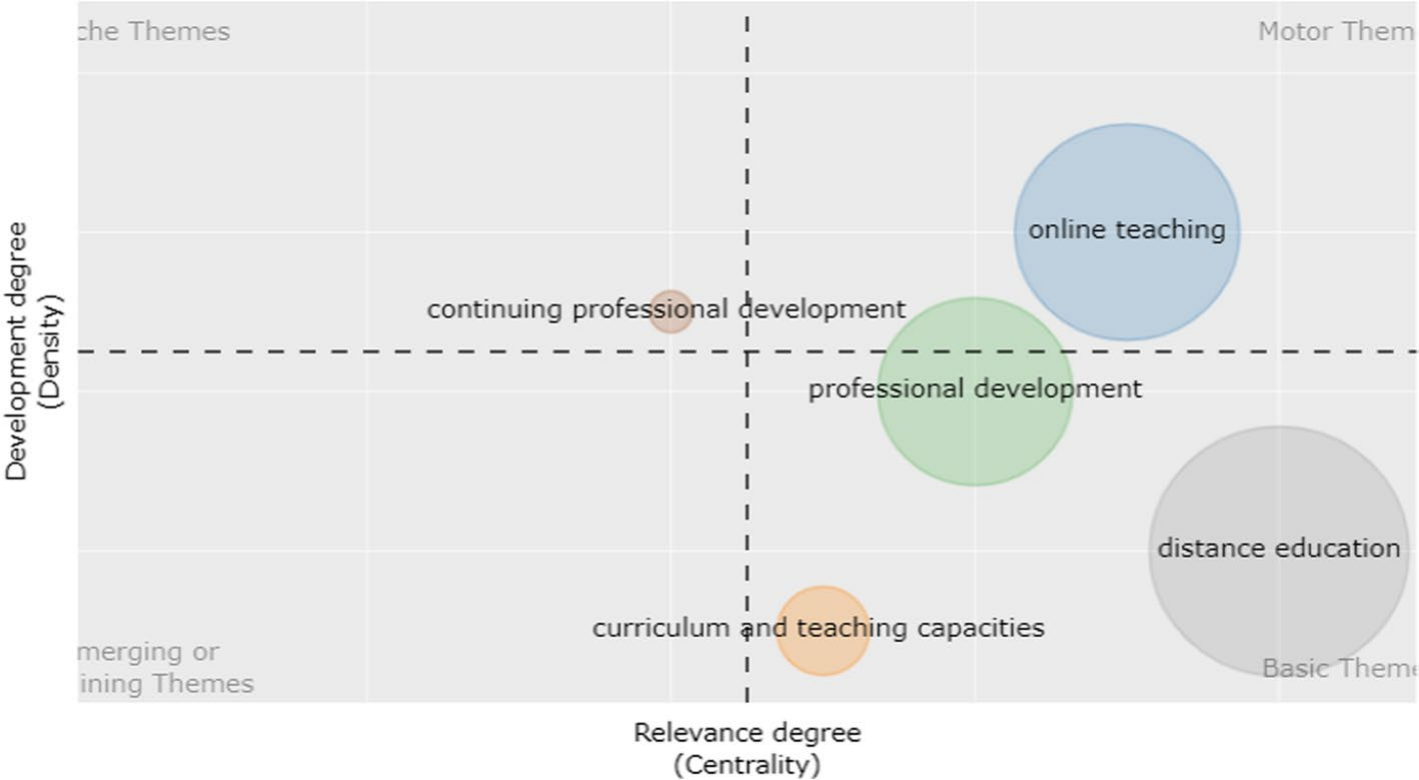
Thematic diagram of 1997–2008

The five main themes in time slice 1 (1997–2008) are presented in Fig. 6. Online teaching was taken as a well-developed and important topic for this study field with high centrality and high density in the upper-right quadrant. Professional development, distance education, and continuing professional development in the lower-right quadrant were well-developed general themes, and different domains were included in these topics. There were no emerging or declining themes in this period. Continuing professional development in the upper-left quadrant was taken as a marginal but well-developed topic.

**Fig. 7:**
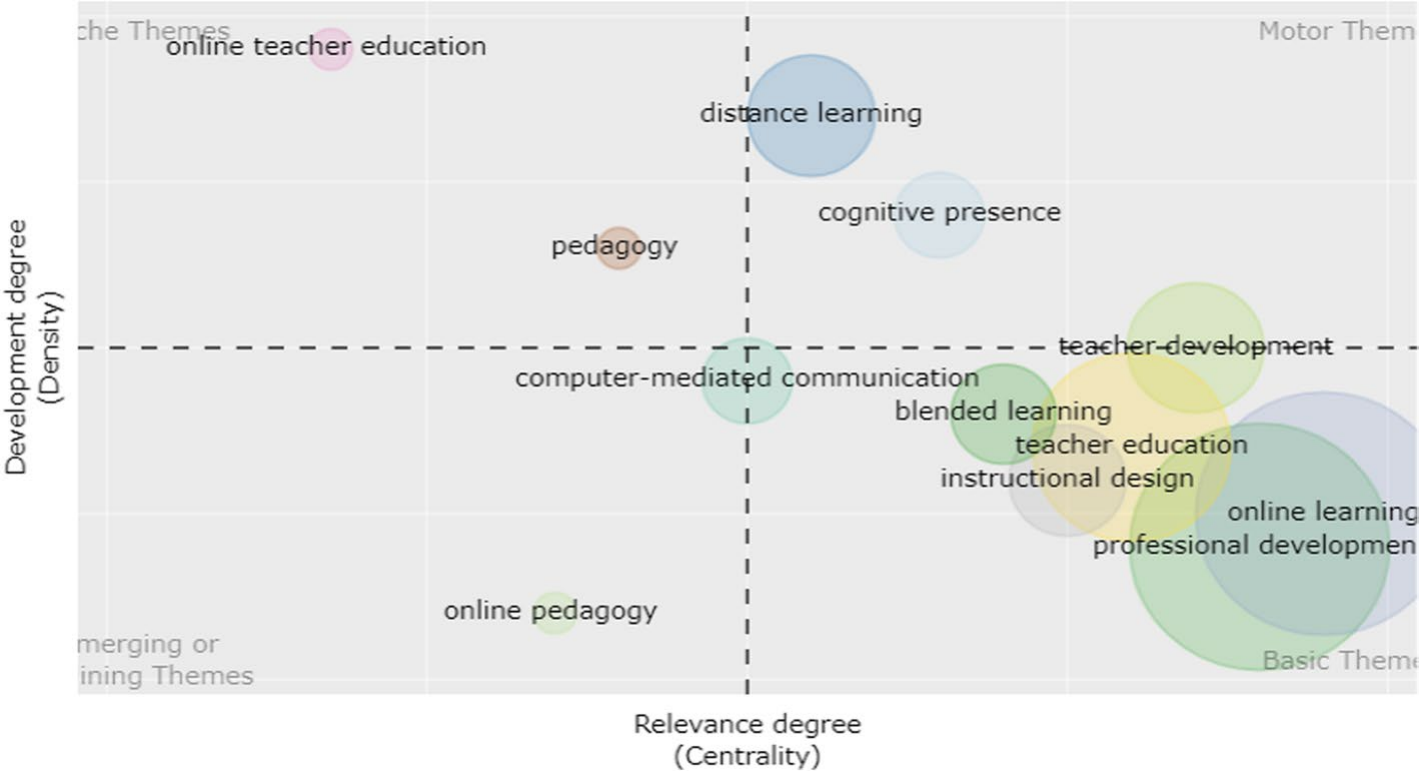
Thematic diagram of 2009–2016

Time slice 2 (2009–2016) showed an obvious change from Time slice 1, with more topics appearing in this stage (Fig. 7). Distance learning and cognitive presence became important motor themes in this period for the whole study. With high centrality and low density, teacher education, professional development in an online context were general study interests for the researchers, and there were different study focuses for each topic. Instructional design became another basic theme in this stage. Studies focusing on online pedagogy appeared as peripheral while the specialized theme and computer-mediated communication shifted from the margin to more centrality in the academic field, as shown clearly in the lower-left quadrant. Online teacher education and pedagogy were presented in the last quadrant as marginal albeit remaining as highly developed topics.

**Fig. 8:**
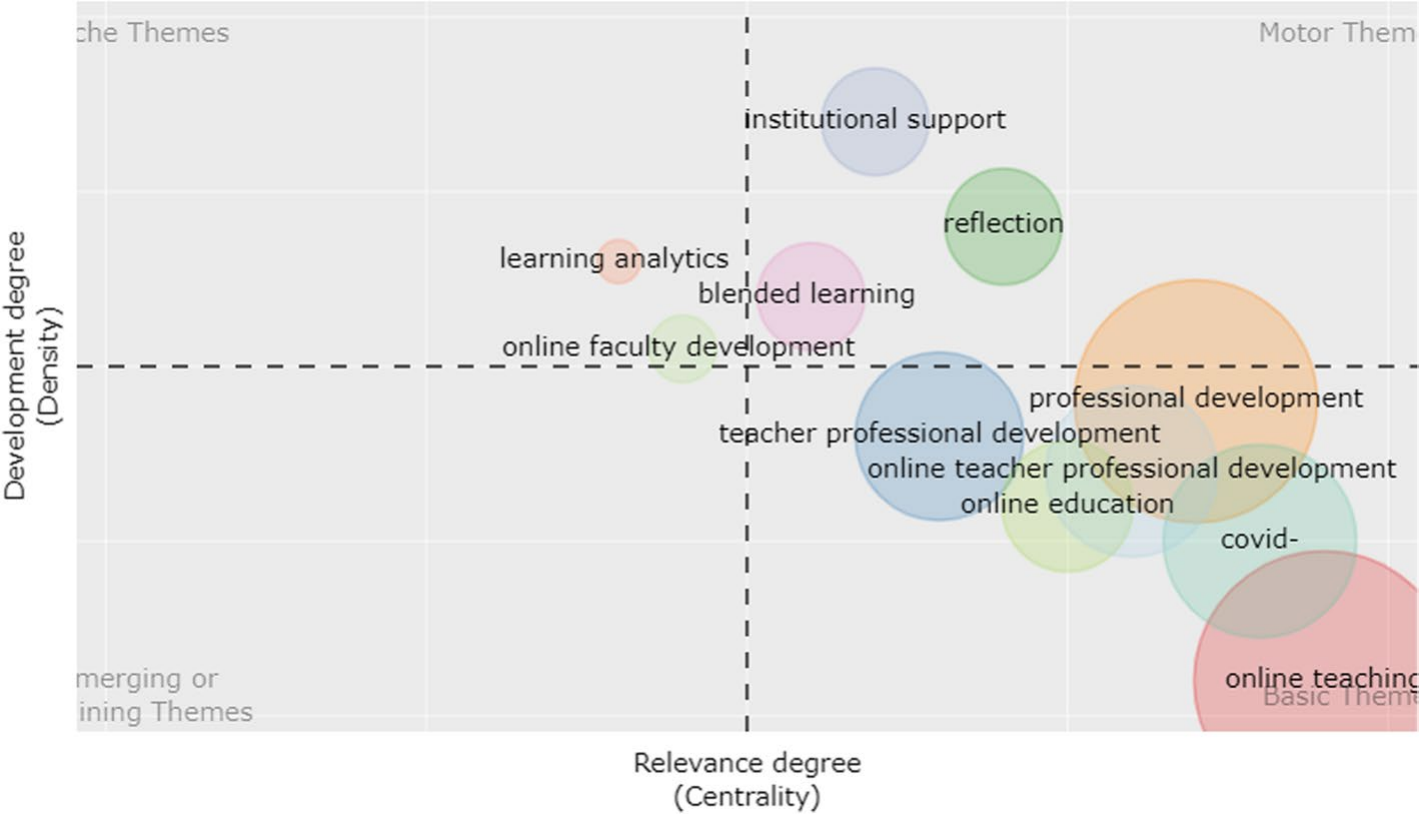
Thematic diagram of 2017–2021

In time slice 3 (2017–2021), institutional support and reflection were the new motor themes together with blended learning, the basic theme in the previous time slice (Fig. 8). Professional development of teachers and other similar expressions were still the transversal themes in recent years as they were in the second period. Some new topics such as Covid-19 emerged as new study hotspots. Online teaching was shifted from a peripheral position in the second stage to a more central position in the third stage as shown in the lower-right quadrant. There was no topic discussed by researchers here with both low centrality and density. In the upper-left quadrant, learning analytics gained more focus from the researchers and appeared as a highly developed topic. Online faculty development became a more peripheral theme in this time slice.

## Bibliometric analysis of literature: discussion

The first research question touched on the distribution of OFPD-related articles. Since publication of the first OFPD article by Briano et al. (1997), the number of publications maintained a steady growth. From a low-production period before 2000, a significant surge of documents occurred from 2009 onward due to the advent of information and communication technology (ICT) and the Internet. The sudden outbreak of Covid-19 at the end of 2019 might be among the main reasons that brought about high numbers of publications in 2020. It is imperative to facilitate teachers to adapt to their new role that requires specific skills in the online environment when the traditional learning mode is not feasible.

In examining the second research question regarding the contributors of OFPD literature, it is worth noting that some journals received higher citations from fewer publications. Concerning the authors’ contributions, Lotka’s law (Lotka, 1926) that was used to evaluate the productivity distribution of the authors showed that the most impactful and relevant authors usually occur in small numbers in any area of study. Northcote, as the most productive author, had a great interest in threshold concepts that represented critical learning stages (Kilgour, et al., 2019). In her studies, Northcote, as well as other authors, did much empirical research to explore how thresholds concepts provided pedagogical guidelines for novice online teachers in professional development programs (Gosselin et al., 2016; Northcote, et al., 2015). These studies are crucial to meeting the ongoing needs of a targeted and responsive curriculum for online academic staff. In considering the impact of the authors, citation analysis was performed to identify the most influential authors in the field of OFPD. In this regard, Shea was the most impactful author with a total of 298 citations. Having started publishing at an earlier time, the studies of Shea were significantly related to the participation of faculty and learners in the online environment (Shea & Bidjerano, 2009), an important topic in the exploration of the early development of online teaching. As to the contribution of various countries, the USA, United Kingdom, and Australia contributed the most in terms of both productivity and citations. Hence, these developed countries had greater voice in the OFPD academic community. At the same time, it is important to observe the emergence of some developing countries in academic discourse concerning OFPD, such as China, India, Turkey, and Malaysia. Given that online education is gaining popularity worldwide, developing countries are beginning to contribute their share to online education and are committed to seeking solutions to the best practices for OFPD. From the perspective of the geographical distribution of OFPD-related publications, America and Europe have played the most active roles, with Asia on the rise in this connection.

Regarding the third research question, seven clusters grouped with the greatest link strength keys were visualized. The latest research theme focused on how professional development helped faculty to conduct online teaching during the global Covid-19 pandemic outbreak which had greatly impacted education throughout the world. Although online education is not new (Ferdig & Kennedy, 2014), many teachers have found themselves unprepared for the challenge when they need to switch to online instruction (Hodges et al., 2020). Therefore, it was imperative for institutions to provide targeted professional development for teachers to shift successfully and smoothly from on-campus teaching to the online mode (Hartshorne et al., 2020). Besides, TPACK has been given much attention by researchers in their quest to explore online faculty professional development. Berry (2019) investigated how professional development provided faculty knowledge in technology, pedagogy, and content in an online program. Berry's (2019) findings indicated the importance of guided practice to improve teachers’ technical knowledge and biweekly meetings with the online community.

The theme “pedagogy” shows the urgent need for pedagogy content for online teachers. As mentioned in previous literature, most novice faculty began their online teaching with little or no training on online delivery (Alexiou-Ray & Bentley, 2015) and minimal training in online pedagogy (Gabriel & Kaufield, 2008). Additionally, faculty pay much more attention to online course design and development than the technical skills needed in online teaching (Taylor & McQuiggan, 2008). Many professional developmental training or programs have been considered to be less valid owing to the emphasis on technology rather than pedagogy (Lane, 2013). Without pedagogical training for online teachers, there will be low-quality online course design and negative faculty online participation, and this will result in a reduction in students’ quality-learning experience and teachers’ satisfaction with online education (Mohr & Shelton, 2017). Indeed, online teachers desire more online pedagogy support to transform themselves as successful online instructors.

Community building is a hot topic discussed in the online setting as online teaching has brought about a sense of isolation both intellectually and socially for online faculty (Baran & Correia, 2014). Research has shown that building a community for online faculty can ease the feeling of isolation and disconnect caused by the online mode (Mohr & Shelton, 2017). Besides that, faculty needs to interact with support personnel and colleagues for assistance and guidance to address the issues encountered in teaching online. Previous studies have shown the contribution of collegial learning groups to faculty’s adaption to online teaching (Baran et al., 2013; Samarawickrema & Stacey, 2007). Moreover, it is necessary to set up a collaborative professional community to cultivate a shared vision among online faculty. The combination of different forms of professional development such as community building, mentoring, and using social media would be critical to the support for online faculty. It is important to create an online community of practice, either focusing on peer support or focusing on social collaboration among different stakeholders, so that the online faculty would be able to function more effectively.

## Limitations and future directions

Though the strict procedure of bibliometric analysis has been followed, some limitations still exist in the study. First of all, since the data were retrieved from a single Scopus database, it was possible to miss some important publications indexed in other databases. Another omission could arise from the fact that only journal articles in English were taken into consideration as the data selection source. Finally, since data mining in the bibliometric analysis is limited to the titles, abstracts, and keywords instead of whole text analysis, some important key concepts and theme developments might vary compared with data mining from the text.

Considering the aforesaid limitations, several suggestions are offered for future studies. Firstly, the data coverage should be widened by integrating the content from different databases such as Web of Science, Google Scholar, and Dimensions so that more comprehensive and complete data would be included in the study. Secondly, future studies could include all publication sources and document types, in case some informative and relevant publications are missed. Thirdly, some other techniques can be used, such as text analysis, to uncover deeper textual information that would enrich research findings on OFPD.

## Conclusion

Substantial changes have been taking place in higher education, especially with regard to online education that is developing at an exceptional pace owing to the Covid-19 pandemic that is still raging. Shifting from traditional face-to-face teaching to the online mode necessitates professional development for these online faculty. Overall, the study results demonstrate the increasing importance of OFPD across the world. Furthermore, discovering and studying major works in this area might help aspiring academics with their research by offering essential information on the subject. It is our hope that this work will contribute significantly to the advancement of this sector, by identifying the field's research potential, the most impactful contributors, and the most common themes.

## Data Availability

Not applicable.

## Abbreviations

OFPD: Online faculty professional development
COVID-19: Coronavirus disease of 2019
CSV: Comma-separated value
PRISMA: Preferred Reporting Items for Systematic Reviews and Meta-Analysis
TPACK: Which stands for technology, pedagogy, and content knowledge
ICT: Information and Communication Technology
